# Does Unstable Employment Have an Association with Suicide Rates among the Young?

**DOI:** 10.3390/ijerph14050470

**Published:** 2017-04-28

**Authors:** Chungah Kim, Youngtae Cho

**Affiliations:** 1 Department of Health Policy, McMaster University, Hamilton, ON L8S 4L8, Canada; kimc19@mcmaster.ca; 2 Department of Public Health Science, School of Public Health, Seoul National University, Seoul 151-172, Korea

**Keywords:** suicide, unstable employment, cross-national study, EPL (employment protection legislation), unemployment

## Abstract

Although a growing body of literature has indicated that unemployment has a positive association with suicide, the dynamic aspects of unstable employment have not yet been considered in suicidology. This study explored the association between employment stability and completed suicide among people aged 25–34 years in 20 OECD (Organization for Economic Cooperation and Development) countries with time-series data (1994–2010). In order to consider the different aspects of unstable employment, we tested the impacts of employment protection legislation indicators as another proxy of job insecurity (employed, but unstable) apart from unemployment rates. Covariates, including economic growth rates, GDP per capita, fertility rates, and divorce rate, were controlled for. The analysis was designed to be gender- and age-specific, where observations with ages of 25–29 were separated from those with ages of 30–34. Random effect models were applied to examine changes over time in suicide rates, and other models were presented to check robustness. The results showed that it is a low level of employment protection, rather than unemployment itself, that was associated with increased suicide rates among all of the studied populations. The magnitude of the effect differed by gender.

## 1. Introduction

Studies exploring the impact of economic determinants of suicide have been conducted for more than a century. Unemployment is one of the most frequently used proxies of these economic determinants because the status of being unemployed implies a loss of income for individuals, and aggregated unemployment rates are associated with decreased levels of national wealth [1,2]. Many studies have suggested that unemployed males have higher suicide rates than employed males, while female suicide rates have shown inconsistent findings [1,3,4,5]. Other studies have failed to find such an association. For example, Andres confirmed a null association between unemployment rates and suicide rates in 15 European countries during the period 1970–1999, conducting fixed effects models analysis, and Neumyer also reported similar results among 68 countries from 1980 to 1999 via fixed and random effects models [6,7]. Nevertheless, the findings of the more recent studies encouraged by the Great Recession in 2007–2008 supported a positive relationship [2,4,8,9,10].

Whether these studies showed a relationship or not, they share a common problem in understanding the nature of unemployment. The problem is that unemployment has been understood as a signifier of the macro-socioeconomic environment. However, unemployment is affected not only by the economic environment, but also by political factors, such as labor market and welfare state policies, and may not correspond to macroeconomic fluctuations [2,11]. In addition, unemployment has tremendous implications on social meanings, such as anxiety over an uncertain future, social exclusion, lowered self-efficacy, and so on, aside from the decreased level of material well-being for each individual [12]. Additionally, it is well known that hopelessness, one of the most direct risk factors for suicide, is closely associated with unemployment [13]. Thus, we need to understand unemployment not just as a proxy of macroeconomic fluctuations, but as an independent concept of employment instability.

Given that we understand unemployment as an independent concept, the dichotomous and static distinction in the traditional understanding of unemployment between employed and unemployed status does not reflect all of the dimensions of employment instability [12,14]. To begin with, unemployed status, itself, can also be divided into different statuses (e.g., long-term and short-term unemployment) [15]. Ignoring the complex, dynamic nature of employment conditions will only identify one side of employment instability. For example, if the quality of employment deteriorates because of an economic recession, unstable employment, such as fixed-term or subcontracted labor, may increase, while unemployment rates decrease [16]. Therefore, in order to examine the impact of unstable employment on suicide, it is necessary to consider further aspects of this multifaceted concept of unstable employment. Indeed, many recent studies have shown relationships between the complex dynamics of unstable employment and different kinds of adverse health outcomes, including mental health outcomes [12,14,15,17].

Another limitation of previous research is that most studies exploring the epidemiology of suicide have been interested either in the general tendency among entire populations, or in particular age groups, mainly youth aged 15–24 and the elderly aged 65 and above, because of high suicide rates in these age groups [18,19]. Existing studies, therefore, provide a less sufficient explanation for suicide in the working population. Suicide in young people aged 25–34, likely to be the most vulnerable group due to the unemployment and job insecurity in high-income countries in recent years, is especially rarely addressed. This is because, in OECD countries with a university enrollment rate of over 60% [20], it is people aged 25–34 who are likely to be seeking to enter the labor market for the first time, and will thus be affected by changes in labor market conditions [21]. Furthermore, people at these ages are more likely to experience life events, such as marriage and childbirth, which are well-known as important moderators of suicide.

Building on the contributions and limitations of previous theories and empirical studies, this paper aims to examine the impact of unstable employment on suicide rates in young people aged 25–34 years old in 20 OECD countries. Specifically, this study will investigate unstable employment by dividing it into two different aspects: unemployment and employment protection for the employed.

## 2. Materials and Methods

### 2.1. Variables and Measurements

This study consists of 20 OECD countries due to the availability of data, and the observation period is 1994–2010. It should be noted that some countries have missing values for some variables (see Table 1 and Table 2). Employment condition variables are divided into three parts: (1) unemployment rate, which can be defined as the share of the labor force without work, but available and seeking employment; (2) strictness of employment protection legislation for regular employment (EPR); and (3) strictness of employment protection legislation for temporary employment (EPT). EPR and EPT are two sub-parts of the indicator of employment protection legislation (EPL) elaborated by the Organization for Economic Cooperation and Development (OECD). EPL is a synthetic conceptual construct to measure the strictness and flexibility of employment protection measures along 21 basic items which are classified in three areas: (i) protection of regular workers against individual dismissal; (ii) regulation of temporary forms of employment; and (iii) additional, specific requirements for collective dismissals [21]. Our variables, (1) EPR and (2) EPT, are equivalent to (i) and (ii), respectively. The indicators are calculated by using the OECD Secretariat’s own reading of statutory laws, case law, collective bargaining agreements and law cases, as well as expert opinion from each country and contributions from officials from OECD member countries. Table 3 demonstrates sub-items, measurements, and weighting schemes of the EPR and the EPT. The indicators range from 0 to 6, and higher scores refer to stricter EPL. Although EPL represents only one dimension of the complex set of factors regarding labor market flexibility, the EPL indicator makes it possible to compare the strictness of employment protection over a longer period of time (e.g., comparison between the mid-1990s and the late 2000s) [21]. For more information on the EPL indicator, please see the Supplementary File 1 (Document S1). 

Data on suicide rates for the gender-specific age groups 25–29 years and 30–34 years were obtained from the Eurostat database, Statistics Korea, and Statistics Japan. Suicide was defined as intentional self-harm on the basis of ICD-10, according to the International Classification for Disease. Table 2 presents average gender-specific suicide rates among the two different age-groups by country.

Building on the economic theory of suicide [22,23], two important economic determinants of suicide (i.e., economic growth rates and real GDP per capita) are controlled in the model. Real GDP per capita is expressed as the natural logarithm due to its large numbers compared to other variables in the models. Likewise, factors that reflect social regulation and integration, namely divorce rates and fertility rates, are introduced according to the sociological theory of suicide developed by Durkheim [24]. All variables, their definitions, and their sources are presented in Table 3. Table 4 displays the summary statistics of each variable by country. The raw data used to calculate these statistics are available in the Supplementary File 2 (Spreadsheet S1).

### 2.2. Analysis

We conducted a random effects analysis after confirming that unobserved country- and time-specific effects that might have an impact are independent of the explanatory variables via the Hausman test [25]. A random effects model allows prediction of the time-invariant effects of each country, and is more efficient than fixed effects models since more degrees of freedom can be used in the estimation [25]. The Supplementary Materials contains the results of the fixed effects model and the generalized estimating equation (GEE) model for the suicide rate for the purpose of comparing and checking the robustness of the results (see Supplementary File 3 Tables S1–S3).

The basic model has the following form:

(1) $${\mathrm{Suicide}\;\mathrm{Rates}}_{i,t}\;=\beta_{1}{\mathrm{Unemployment}\;}_{i,t}+\beta_{2}{\mathrm{EPR}}_{i,t}+\beta_{3}{\mathrm{EPT}}_{i,t}\;\;+\beta_{13}\;\left({\mathrm{Unemployment}}_{i,t}\;\times {\mathrm{EPT}}_{i,t}\right)\;+{\;Z}_{i,t}\delta \;+\alpha_{i}+\varepsilon_{i,t}$$

The subscripts *i* = 1, 2, …, 20 and *t* = 1994, 1995, …, 2010 indicate each country and time period, respectively; $\beta_{1},\;\beta_{2},$ and $\beta_{3}$ are estimated coefficients of ${\mathrm{Unemployment}}_{i,t}$, ${\mathrm{EPR}}_{i,t}$, and ${\mathrm{EPT}}_{i,t}$, respectively; ${\mathrm{Unemployment}}_{i,t}\times {\mathrm{EPT}}_{i,t}$ is an interaction term to examine whether the interaction between unemployment and temporary employment practice affects the results; $\beta_{13}$ is the estimated coefficient; $Z_{i,t}$ denotes a vector of economic and sociological covariates; ${\delta \;\mathrm{is}\;\mathrm{the}\;\mathrm{estimated}\;\mathrm{coefficient}\;\mathrm{vector};\;\alpha }_{i}$ denotes the random effects; and $\varepsilon_{i,t}$ is a random disturbance term. All statistical analysis was conducted by SAS 9.3 (SAS Institute, Cary, NC, USA)) with a 10% level of statistical significance.

## 3. Results

Table 5 shows correlation coefficients among independent variables and their statistical significance. All coefficients are below 0.5. Proportions of variation among the independent variables were also computed for the four different gender and age groups, but the results were all below 0.6 and most of them were below 0.3 (results not shown here). These results indicate that multicollinearity does not exist among the variables employed in the current analysis [26].

Table 6 demonstrates the results of the random effects model analysis for male and female suicide rates by two age groups (25–29 and 30–34).

The results of all of the models show that the suicide rates of all the age and gender groups in each country are consistently regressed on EPR. Unemployment rates, by contrast, turn out not to be significant and, indeed, the pattern of coefficients are inconsistent. The negative coefficient associated with EPR can be interpreted as follows: after a country lowers the level of employment protection for regular contracts, the suicide rates for both males and females aged 25–34 in the country increase. Notably, the coefficient magnitudes of EPR are higher for males than for females, and higher for males aged 30–34 than for males aged 25–29. Other models confirm these trends, despite the different size of their coefficients. EPT also has a negative impact on suicide rates for males aged 30–34 in both random and fixed effects models.

We now turn to describing the findings for the economic and social variables. To begin with, GDP per capita is consistently negatively associated with suicide rate in all of the studied populations, and the impacts are stronger for males than for females, and for males aged 30–34 than for males aged 25–29, as was the pattern for EPR. These results imply that an increase in the real income in a country has an association with a subsequent decrease in suicide rates. Even though the results of the random effects model did not show significance among females, the coefficient estimates show a consistent negative direction. As for the social variables representing the degree of social integration and regulation, we can find a tendency in all of the different models for male suicide rates to be more regressed on divorce rates, as opposed to female suicide rates, which are more affected by fertility rates, despite the exception of the male (25–29) suicide rates, as shown in Table 6.

## 4. Discussion

### 4.1. What This Study Adds

The findings of this study reveal that lower levels of employment protection for regular contracts had a consistently negative impact on suicide rates among people aged 25–34, whereas unemployment rates did not affect suicide rates in any population, regardless of the model. In addition, we also found that the size of the impact of employment protection varied by gender. Gender differences also existed in the impact of GDP per capita, divorce rates, and fertility rates.

### 4.2. Why Did EPR Matter, and Not Unemployment?

As explained in the introduction, the overall results of previous studies investigating the impacts of unemployment on suicide, despite the heterogeneity of the findings, supported the hypothesis that unemployment is one of the most important socioeconomic factors influencing suicide. This tendency is especially well substantiated by studies that employed individual-level data. For instance, Blackery and Kposawa reported that unemployed individuals died by suicide at rates 2.34 times, and even up to 25.19 times, higher than the employed in several years in New Zealand and the United States, respectively [18,27]. The results of studies using registry datasets in Sweden, Denmark, and Norway were no different [28,29,30]. Given that, why did unemployment not show any impact on suicides either in this study, or in some previous studies that conducted analyses at the aggregate level? One possible answer is that macro unemployment rates, by their very definition, have limitations in showing the actual figure of the employed. The definition of macro unemployment rates excludes groups of the population out of the labor market, such as students, housekeepers, part-time workers, and those who have given up seeking jobs. This exclusion would cause systematic bias in the impact of unemployment rates on suicide rates at the aggregate level because these population groups are not distinguishable from the unemployed [31], resulting in underestimating the size of the unemployed population. This problem could be especially salient in the age groups 25–34. People in this age group often remain in student status, choose to be unpaid household workers immediately after getting married, and work part-time after childbirth more often than do people in other age groups. This hypothesis can be substantiated by the above cohort studies, which reported a strong association between the non-active labor force and suicide for male and female adults [18,27]. The different size of the bias (depending on the cultural context) would have caused the inconsistent results found in studies that analyzed unemployment and suicide at the aggregate level. 

There are two provisional reasons why EPR, rather than unemployment, had an impact on suicides. To begin with, EPR is a more precise predictor of the actual figure of unemployment. As opposed to aggregate unemployment rates, which systematically exclude the real unemployed, EPR better reflects changes in the laws and policies that govern labor markets. These changes affect labor market flexibility and the actual size of the unemployed population. EPR, which takes these laws and policies into account is, therefore, a less biased measurement method. In addition to better predicting the real unemployment rates, EPR captures the multidimensional dynamics of unstable employment. A decrease in EPR may indicate an increase in job insecurity because of easier layoff procedures and less regulation, an increase in economic uncertainty because of decreased severance pay, and an increase in unemployment shocks due to a shortened period of required notice of dismissal for workers [32]. These increases are closely associated with the aggravated mental status that may have an impact on suicide rates. EPT, despite being significant only in male (30–34) suicide rates, also demonstrated robust estimation in all studied populations, since changes in EPT also predict the number of temporary workers in a society by means of changes in laws and policies regulating the use of temporary employment. Nevertheless, EPT has a less significant impact on suicide rates, probably because the definitions of temporary, subcontracted, non-standard, and atypical workers (i.e., precarious employment) are very heterogeneous in each country and are more likely to be affected by the historically unique characteristics of the individual labor market policies in a given country [14]. As a result, EPT may underestimate the detrimental impacts of precarious employment. Moreover, even though EPR and EPT measure regulation of regular and temporary employment, respectively, they are so deeply intertwined that both, together, indicate the degree of unstable employment status while being employed [32]. These dynamics should be more accurately addressed in future studies using individual-level data.

### 4.3. Gender Differentials in the Impacts of Unstable Employment, Income, and Sociological Variables

One of the most consistent trends in all of the results of the three different models is that unstable employment has a stronger impact on men, and that men in their early 30s are even more affected. This can be seen clearly from the size of the EPR coefficients, but it is also evident in that the EPT was significant only in men aged 30–34. The pattern of this trend is equally represented in GDP per capita, which is a proxy of real income for individuals. These results are consistent with previous studies which reported that male suicide was more sensitive to unemployment and low income levels [11,33]. The results can be explained by the ‘male breadwinner system’, in which males are still under the burden of the role of the primary breadwinner, so that unemployment and loss of income imply not only economic hardship, but also a failure to live up to family responsibilities and the masculine role. This brings about increased suicidal behaviors and these suicidal behaviors are more likely to result in death than for women because men are likely to choose more lethal means of self-harm [34]. The theory can also offer an explanation for the result that the impacts of unstable employment and income on suicide were the strongest among males aged 30–34, as members of that population are more likely to be married than are males aged 25–29 and are, therefore, more exposed to the role of breadwinner.

Finally, the gender differentials in the impacts of divorce and fertility rates on suicide found in the results also supported existing studies. Durkheim reported that, while both being married and having children protected males from suicide via increased social integration and regulation, women were less likely to be affected by marital status. In some populations, women were even protected by being unmarried, probably due to the patriarchal nature of marriage [24]. However, fertility rates had a strong negative impact on female suicide in this study. It is also well aligned with Durkheim’s hypothesis that parenthood protects women from suicide because of their expected role of caring for a child within a household. Empirical studies that tested this hypothesis with individual-level data in Norway reported the same results [35,36]. Additionally, since the protective effect may vary at different stages of life (i.e., having children protects females from suicide when they are caring for their children), it is plausible that our female study population, consisting of women in their childbearing years, would be sensitive to the fertility rates [36]. As such, gender differentials in the effects of both unstable employment and confounding factors on suicide can be simultaneously understood by appealing to gender role stereotypes.

### 4.4. Limitation

Four main limitations of this paper can be noted. First, there were some missing observations in suicide rates in some countries (Belgium, France, Italy, Poland, and Slovakia), which might have an impact on the results. However, it is argued that the missing observations did not have a systematic pattern in this study and are not widespread enough to have a significant impact on the results. Secondly, this study takes an ecological design. Ecological study designs not only prevent results from the aggregated data from being inferred as causal relationships on the individual level, but also make it impossible to differentiate compositional effects from contextual impacts. Nevertheless, ecological research can offer important provisional research questions that have not yet been answered, and the results of this study are meaningful in the sense that we explored the relationships between multidimensional aspects of unstable employment and suicides. Thirdly, important covariates regarding mental and physical health status at the population level were omitted in our model. This is because we agree that mental health status and socioeconomic factors do not have separate and independent effects and, thus, mental health status can be understood as an intermediator [15]. According to recent studies [37,38,39], unemployment and low levels of income are important predictors of non-adherence to treatment medications among patients with mental disorders. As such, it shows a possible facet of the intermediate relationship between helplessness caused by unstable employment and subsequent impaired mental status. Finally, due to the nature of the EPL indicator, which is measured by laws and policies, the impact of unstable employment might not be accurately estimated. However, changes in the EPL indicator can still reflect the degree of job insecurity in the absence of valid available variables for cross-national studies. Future research can achieve greater rigor and relevance by taking cohort and case-control designs to address the relationship between unstable employment and suicide by death at the individual level.

## 5. Conclusions

Unstable employment had a significant impact on suicide among people aged 25–34. In general, this study showed that macro unemployment rates have limitations in predicting suicide rates, and that changes in EPL, instead, capture the dynamics of employment status associated with suicide rates. Economic factors, especially decrease in GDP per capita, also turned out to be a good predictor of increased suicide rates. The impacts of EPL and GDP per capita on suicide rates were different by gender. Divorce rates and fertility rates also affected suicide rates. The findings of this study have implications for policy-makers trying to come up with a suicide prevention policies for young people: increases in job security, decent levels of income, and family support for young people would be effective means of achieving this goal.

## Figures and Tables

**Table 1:** Gender- and age-specific average suicide rates of 20 OECD countries (1994–2010).

Country	Male Suicide Rates		Female Suicide Rates	
	25–29	30–34	25–29	30–34
Austria	22.63	25.19	5.34	6.76
Belgium	28.4	31.4	7.91	9.01
Czech Republic	21.45	23.73	3.31	4.12
Denmark	16.6	18.76	4.09	4.66
Finland	41.55	42.53	10.49	11.17
France	20.47	27.54	5.03	7.24
Germany	16.39	17.74	4.11	4.53
Greece	6.35	5.78	1.16	1.53
Hungary	27.5	39.29	4.97	7.65
Italy	9.86	10.65	2.34	2.78
Japan	25.36	27.24	10.9	11.35
The Netherlands	13.03	15.53	4.93	6.13
Poland	23.96	27.4	2.63	3.55
Portugal	8.8	9.55	1.81	2.69
Slovakia	16.75	21.18	1.73	2.34
South Korea	19.71	22.66	12.68	11.95
Spain	11.19	12.09	2.49	2.90
Sweden	17.01	18.17	6.07	7.05
Switzerland	24.85	21.38	6.94	7.29
UK	16.68	18.12	3.63	3.79
**Mean**	19.41	21.67	5.17	5.94
**S.D.**	8.14	9.33	3.23	3.17

**Table 2:** Variable definitions and data sources.

Variable	Definition	Data Source	Observation
Suicide rate	Intentional self-harm (annual deaths per 100,000)	Europe: Eurostat Japan: Statistics Japan **^1^** Korea: Statistics Korea **^2^**	323
Unemployment rate	% of the labor force without work but available and for seeking employment	World Bank	340
EPR	Strictness of employment protection legislation for regular employment	OECD	340
EPT	Strictness of employment protection legislation for temporary employment	OECD	340
Economic growth rate	Annual percentage growth rate of GDP at market prices based on constant local currency	World Bank	340
GDP per capita	Purchasing power parity, dollars	World Bank	340
Divorce rate	Number of divorces per 1000 people	OECD	340
Fertility rate	Age-specific birth rates over all reproductive ages	United Nations (UN)	340

**^1,2^** Data of Japan and Korea are not available on the Eurostat database.

**Table 3:** Measurement schemes of the EPL.

Basic Item	Short Description		Assignment of Numerical Scores						
			Assigned Scores						
			0	1	2	3	4	5	6
**Individual dismissals of workers with regular contracts**									
**Item1** Notification Procedures	**Scale 0–3** 0 when an oral statement is enough; 1 when a written statement of the reasons for dismissal must be supplied to the employee; 2 when a third party (such as works council or the competent labor authority) must be notified; 3 when the employer cannot proceed to dismissal without authorization from a third party;		Scale (0 − 3) × 2						
**Item2** Delay involved before notice can start	**Days** Estimated time includes, where relevant, the following assumptions: six days are counted in case of a required warning procedure; one day when dismissal can be notified orally or the notice can be directly handed to the employee; two days when a letter needs to be sent by mail; and three days when this must be a registered letter.		≤2	<10	<18	<26	<35	<45	≥45
**Item3** Length of the notice period at	9 months tenure	**Months**	0	≤0.4	≤0.8	≤1.2	≤1.6	<2	≥2
	4 years tenure	**Months**	0	≤0.75	≤1.25	<2	<2.5	<3.5	≥3.5
	20 years tenure	**Months**	<1	≤2.75	<5	<7	<9	<11	≥11
**Item4** Severance pay at	9 months tenure	**Months pay**	0	≤0.5	≤1	≤1.75	≤2.5	<3	≥3
	4 years tenure	**Months pay**	0	≤0.5	≤1	≤2	≤3	<4	≥4
	20 years tenure	**Months pay**	0	≤3	≤6	≤10	≤12	≤18	>18
**Item5** Definition of justified or unfair dismissal	**Scale 0–3** 0 when worker capability or redundancy of the job are adequate and sufficient ground for dismissal; 1 when social considerations, age, or job tenure must when possible influence the choice of which worker(s) to dismiss; 2 when a transfer and/or a retraining to adapt the worker to different work must be attempted prior to dismissal; 3 when worker capability cannot be a ground for dismissal;		Scale (0 − 3) × 2						
**Item6** Length of trial period	**Months** Period within which, regular contracts are not fully covered by employment protection provisions and unfair dismissal claims can usually not be made.		≥24	>12	>9	>5	>2.5	≥1.5	<1.5
**Item7** Compensation following unfair dismissal	**Months pay** Typical compensation at 20 years of tenure, including back pay and other compensation (e.g., for future lost earnings in lieu of reinstatement or psychological injury), but excluding ordinary severance pay.		≤3	≤8	≤12	≤18	≤24	≤30	<30
**Item8** Possibility of reinstatement following unfair dismissal	**Scale 0–3** 0 no right or practice of reinstatement; 1 reinstatement rarely or sometimes made available; 2 reinstatement fairly often made available; 3 reinstatement (almost) always made available;		Scale (0 − 3) × 2						
**Item9** Maximum time to make a claim of unfair dismissal	**Months** Maximum time period after dismissal notification up to which an unfair dismissal claim can be made.		Before dismissal takes effect	≤1	≤3	≤6	≤9	≤12	>12
**Temporary employment**									
**Item10** Valid cases for use of fixed-term contracts (FTC)	**Scale 0–3** 0 fixed-term contracts are permitted only for “objective” or “material situation”, i.e., to perform a task which itself is of fixed duration; 1 if specific exemptions apply to situations of employer need (e.g., launching a new activity) or employee need (e.g., workers in search of their first job); 2 when exemption exist on both the employer and employee sides; 3 when there are no restrictions on the use of fixed-term contracts.		6 − (Scale (0 − 3) × 2)						
**Item11** Maximum number of successive FTC	**Number**		No limit	≥5	≥4	≥3	≥2	≥1.5	<1.5
**Item12** Maximum cumulated duration of successive FTC	**Months**		No limit	≥36	≥30	≥24	≥18	≥12	<12
**Item13** Types of work for which temporary. Work agency (TWA) employment is legal	**Scale 0–4** 0 when TWA employment is illegal; 1 only allowed in specified industries; 2 only allowed for “objective reasons”; 3 generally allowed, with specified exceptions; 4 generally allowed, no (or minimal) restrictions.		6 − (Scale (0 − 4) × 6/4)						
**Item14** Restrictions on number of renewals	**Yes/No**		-	-	No	-	Yes	-	-
**Item15** Maximum cumulated duration of TWA assignments	**Months**		No limit	≥36	≥24	≥18	≥12	>6	≤6
**Item16** Does the set-up of a TWA require authorization or reporting obligations	**Scale 0–3** 0 no authorization or reporting requirements; 1 requires special administrative authorization; 2 requires periodic reporting obligations; 3 both authorization and reporting requirements.		Scale (0 − 3) × 2						
**Item17** Do regulations ensure equal treatment of regular and agency workers at the user firm?	**Scale 0–2** 0 no requirement for equal treatment; 1 equal treatment regarding pay or working conditions; 2 equal treatment regarding pay and working conditions.		Scale (0 − 2) × 3						
**Strictness of employment protection—Individual dismissals (Regular workers), summary indicator weights**									
**Summary Index of EPR** **Scale 0–6**	**Sub-Indexes** **Scale 0–6**		**Items** **Scale 0–6**						**Weights**
Individual dismissals—Regular worker (EPR)	Procedural inconveniences (1/3)		1. Notification procedures						(1/2)
			2. Delay to start a notice						(1/2)
	Notice and severance pay for no-fault individual dismissals (1/3)		3. Notice period after:						
			9 months						(1/7)
			4 years						(1/7)
			20 years						(1/7)
	Difficulty of dismissal (1/3)		4. Severance pay after:						
			9 months						(4/21)
			4 years						(4/21)
			20 years						(4/21)
			5. Definition of unfair dismissal						(1/5)
			6. Trial period						(1/5)
			7. Compensation						(1/5)
			8. Reinstatement						(1/5)
			9. Maximum time for claim						(1/5)
**Strictness of employment protection—temporary contracts**													
**Summary index of EPT** **Scale 0–6**	**Sub-indexes** **Scale 0–6**		**Items** **Scale 0–6**						**Weights**
Temporary contracts (EPT)	Fixed term contracts (1/2)		10. Valid cases for use of fixed-term contracts						(1/2)
			11. Maximum number of successive contracts						(1/4)
			12. Maximum cumulated duration						(1/4)
	Temporary work Agency employment (1/2)		13. Types of work for which is legal						(1/3)
			14. Restrictions on number of renewals						(1/6)
			15. Maximum cumulated duration						(1/6)
			16. Authorization and re porting						(1/6)
			17. Equal treatment						(1/6)

**Table 4:** Summary statistics of variables.

Country	Unemployment	EPR	EPT	Economic Growth Rate	GDP per Capita	Divorce Rate	Fertility Rate
Austria	4.176	1.343	0.875	2.118	33,324.32	2.362	1.391
Belgium	8.206	1.801	2.904	1.998	31,733.53	2.876	1.693
Czech Republic	6.541	3.246	0.732	3.104	10,497.92	3.049	1.276
Denmark	5.347	2.138	1.478	1.722	41,431.01	2.662	1.790
Finland	10.088	2.259	1.489	1.722	32,305.63	2.604	1.788
France	10.018	2.391	3.625	1.717	29,994.99	2.085	1.884
Germany	8.753	2.757	1.794	1.375	31,297.87	2.336	1.341
Greece	9.900	2.802	3.926	2.490	17,920.63	1.062	1.343
Hungary	7.965	2.004	0.831	2.319	8108.62	2.437	1.359
Italy	9.435	2.762	3.007	1.087	26,280.99	0.699	1.291
Japan	4.306	1.624	1.129	0.884	36,172.79	1.983	1.359
The Netherlands	4.259	2.867	1.067	2.348	33,888.41	2.099	1.681
Poland	13.724	2.230	1.132	4.625	6813.16	1.353	1.392
Portugal	6.635	4.498	2.699	1.969	15,577.21	1.925	1.420
Slovakia	14.582	2.359	1.243	4.564	9166.71	1.929	1.341
South Korea	3.665	2.526	2.419	4.835	14,083.17	2.450	1.332
Spain	15.112	2.427	3.221	2.730	21,370.73	1.171	1.278
Switzerland	3.653	1.595	1.125	1.731	48,437.18	2.370	1.458
Sweden	7.424	2.660	1.386	2.800	35,859.92	2.349	1.722
U.K.	6.247	1.139	0.309	2.584	30,835.03	2.744	1.769
**Minimum**	2.000	1.032	0.250	−6.854	2812.60	0.500	1.076
**Maximum**	23.900	4.583	4.750	10.494	70,370.02	3.800	2.030
**Mean**	8.002	2.371	1.820	2.436	25,754.99	2.127	1.495
**S.D.**	4.054	0.732	1.157	2.590	13,893.70	0.665	0.228

**Table 5:** Correlation matrix.

Explanatory Variables	Unemployment	EPR	EPT	Economic Growth Rate	GDP per Capita	Divorce Rate
EPR	0.125					
	−0.021					
EPT	0.282	0.353				
	(<0.0001)	(<0.0001)				
Economic growth rate	0.08	0.049	−0.002			
	−0.141	−0.371	−0.973			
GDP per capita	−0.430	−0.352	−0.138	−0.369		
	(<0.0001)	(<0.0001)	−0.011	(<0.0001)		
Divorce rate	−0.406	−0.244	−0.488	−0.059	0.243	
	(<0.0001)	(<0.0001)	(<0.0001)	−0.277	(<0.0001)	
Fertility rate	−0.198	−0.190	−0.070	−0.141	0.501	0.325
	0	0	−0.198	−0.009	(<0.0001)	(<0.0001)

Numbers in parentheses are *p*-values.

**Table 6:** Random effects model for gender- and age- specific suicide rates.

Explanatory Variables	Dependent Variables			
	Male Suicide Rates		Female Suicide Rates	
	25–29	30–34	25–29	30–34
Unemployment	0.249	−0.052	−0.079	−0.027
	−0.184	−0.186	−0.101	−0.81
EPR	−4.229 ***	−5.997 ***	−2.613 ***	−2.381 ***
	−1.422	−1.464	−0.799	−0.738
EPT	−1.214	−1.650 *	−0.658	0.064
	−0.905	−0.915	−0.5	−0.483
Unemployment × EPT	−0.019	0.047	0.042	0.003
	−0.077	−0.077	−0.042	−0.041
Economic growth rate	0.118	−0.053	−0.066	−0.052
	−0.094	−0.186	0	−0.081
GDP per capita	−2.287 *	−4.450 ***	−0.182	−0.023
	−1.183	−0.885	−0.483	−0.627
Divorce rate	1.697 *	3.113 ***	−0.475	0.781 *
	−0.873	−0.885	−0.483	−0.464
Fertility rate	−0.610	1.877	−4.330 ***	−2.948 ***
	−2.252	−2.282	−1.246	−1.198
Intercept	52.498 ***	75.769 ***	22.032 ***	15.135 **
	−12.1	−12.304	−6.717	−6.402
Observations	323	323	323	323

Numbers in parentheses are robust standard errors. The number of observations is 323 instead of 340, as would be expected given 20 countries and a period of 17 years (1994–2000), due to missing observations for some countries. * *p* < 0.10; ** *p* < 0.05; *** *p* < 0.01.

## Supplementary Materials

The following are available online at www.mdpi.com/1660-4601/14/5/470/s1, Document S1: Calculating summary indicators of EPL, Spreadsheet S1: Data tables used for analysis, Table S1: Fixed effects model for gender- and age- specific suicide rates, Table S2: Random effects model for gender- and age- specific suicide rates, Table S3: Generalized estimating equation model for gender- and age- specific suicide rates.
